# Development of Silk Fibroin-Based Sponges Loaded with LL-37-Derived Peptides for the Control of Orthopedic Infections

**DOI:** 10.3390/ijms26167775

**Published:** 2025-08-12

**Authors:** Vincenzo Pennone, Giada Meogrossi, Giacomo Carenzi, David Sarlah, Marco Biagiotti, Arianna B. Lovati

**Affiliations:** 1 Cell and Tissue Engineering Laboratory, IRCCS Istituto Ortopedico Galeazzi, via Cristina Belgioioso 173, 20157 Milan, Italy; arianna.lovati@grupposandonato.it; 2 Fondazione Istituto Insubrico Ricerca per la Vita, via Lepetit 34, 21040 Gerenzano, Italy; giadameogrossi@ricercaperlavita.it (G.M.); giacomocarenzi@ricercaperlavita.it (G.C.); 3 Department of Chemistry, Rice University, Houston, TX 77005, USA; david.sarlah@unipv.it; 4 Department of Chemistry, University of Illinois at Urbana-Champaign, Urbana, IL 61801, USA; 5 Department of Chemistry, University of Pavia, Viale Taramelli 12, 27100 Pavia, Italy; 6 KLISBio srl, Via Ludovico Ariosto, 21, 20091 Bresso, Italy; m.biagiotti@klis.bio

**Keywords:** antimicrobial peptides, FK-16, GF-17, silk fibroin, orthopedic infections, methicillin-resistant *Staphylococci*, local antimicrobial delivery system

## Abstract

*Staphylococcus* species are often the cause of implant-related infections, posing a significant clinical challenge in orthopedics. Antimicrobial peptides (AMPs) like LL-37-derived FK-16 and GF-17 offer promising alternatives to conventional antibiotics; however, they require suitable delivery systems to overcome rapid degradation. The aim of this study was to develop and evaluate silk fibroin (SF) and osteoinductive peptide-enriched silk fibroin (PSF) sponges that can be used locally for FK-16 and GF-17 delivery. Two concentrations of FK-16 or GF-17 were loaded into SF and PSF sponges. Swelling behavior and AMP release profiles were analyzed for 72 h. Time-kill assays were conducted on MRSE and MRSA clinical strains to assess antimicrobial activity. FK-16 released quickly (>90% within 24 h) and then maintained a stable plateau from both SF and PSF matrices, which was associated with bactericidal activity against MRSE strains. In contrast, the release efficiency of GF-17 was lower and did not achieve significant antimicrobial effects. Neither peptide exhibited effective activity against MRSA under the tested conditions. PSF sponges showed higher swelling and enhanced FK-16-mediated antibacterial performance compared to SF counterparts. FK-16-loaded PSF sponges are a promising biomaterial for treating local orthopedic infections related to MRSE. The findings underscore the significance of peptide–matrix interactions in determining therapeutic outcomes and suggest the need for more in vivo evaluation of AMP-functionalized PSF scaffolds.

## 1. Introduction

Despite the clinical benefits of orthopedic implants, their increasing use has led to a higher incidence of implant-associated infections, which remain a major challenge in surgical practice [1]. The capability of *S. aureus* and *S. epidermidis* to form biofilms significantly impairs host immune responses and limits antibiotic efficacy, contributing to the persistence of infection and the emergence of antimicrobial resistance [2]. Persisting implant-associated infections not only compromise surgical outcomes but also impose a substantial burden on healthcare systems due to the need for revision surgeries and extended patient care [3].

The protective nature of bacterial biofilms and limited blood supply at the implant interface may prevent systemic antibiotics from eradicating the infection [4]. This highlights the pressing need for local antimicrobial approaches to simultaneously control infection and promote tissue repair, particularly in the challenging environment surrounding orthopedic implants [5].

In this context, antimicrobial peptides (AMPs) have emerged as promising alternatives to traditional antibiotics. AMPs exhibit broad-spectrum antibacterial activity, with fast bactericidal mechanisms mainly through membrane disruption, and a lower chance of inducing resistance [6]. Among these, FK-16 and GF-17, two truncated analogs derived from the human cathelicidin LL-37, have shown potent efficacy against a range of Gram-positive pathogens implicated in orthopedic infections, including MRSA and MRSE strains [7]. Importantly, FK-16 and GF-17 exhibit low cytotoxicity towards mammalian cells and minimal hemolytic activity at therapeutic concentrations [7].

In vitro studies have confirmed that FK-16 and GF-17 inhibit bacterial growth and disrupt established biofilms, which is a critical advantage for managing chronic implant-associated infections [8]. The potential for long-term use of these peptides was highlighted by the absence of significant resistance development after repeated bacterial exposure to these peptides [7]. Clinical applications of AMPs are hampered by their sensitivity to proteolytic degradation and rapid clearance from the infection site. To address these challenges, biomaterial-based delivery systems have been explored to enhance the stability and stable release of AMPs [9]. The use of AMPs in biomaterial scaffolds is a novel approach to providing both structural support and localized antimicrobial protection [2]. Silk fibroin (SF), a natural fibrous protein derived from *Bombyx mori* cocoons, has gained attention as a scaffold material due to its biocompatibility, mechanical strength, biodegradability, and ability to be processed into various formats such as films, sponges, and hydrogels [10,11]. SF can protect encapsulated bioactive molecules from degradation and release them in a controlled manner, which is particularly advantageous in orthopedic applications [12]. The enrichment of SF with anionic fibroin-derived polypeptides in Peptide-enriched silk fibroin (PSF) scaffolds has led to osteoinductive properties and the nucleation and growth of apatite [13]. Silk fibroin composites that contain antibiotics have been tested recently to fight implant-related infections, and they have been successful in both in vitro and in vivo models [14,15]. Conventional antibiotics come with risks, such as cytotoxic effects at high local concentrations and the chance of encouraging antibiotic resistance [16]. To address these limitations, the incorporation of AMPs into SF scaffolds has emerged as a promising strategy for orthopedic applications. Controlled release profiles can be achieved by engineering SF-based scaffolds to maintain therapeutic concentrations of AMPs at the target site for extended periods. A 3D scaffold that is made up of SF, chitosan, nano-hydroxyapatite, and LL-37 has been created, which has both antimicrobial and osteoinductive properties. The ability of this construct to inhibit *Staphylococcus aureus* and *Pseudomonas aeruginosa* meant that it could promote bone regeneration in vivo. These multifunctional scaffolds have the potential to prevent infections and enhance tissue integration in orthopedic implants [17]. Antibiotic-loaded SF nanoparticles are effective in delivering antibiotics locally and promoting bone regeneration on titanium implant surfaces [18]. The purpose of this study was to evaluate the antimicrobial effectiveness of SF-based scaffolds loaded with FK-16 and GF-17, based on these advancements. Our goal was to develop a delivery system that could sustain antimicrobial activity while supporting tissue regeneration by optimizing the loading concentrations and release kinetics. Specifically, in this study, we developed SF and osteoinductive peptide-enriched SF sponges loaded with FK-16 and GF-17 to assess their antibacterial efficacy against clinical isolates of methicillin-resistant *Staphylococcus aureus* (MRSA) and *Staphylococcus epidermidis* (MRSE). We also evaluated the peptide release profile and swelling behavior of the delivery systems. Unlike previous work on antibiotic-loaded scaffolds, our approach focuses exclusively on AMPs to highlight their potential as non-antibiotic alternatives, minimizing the risk of antimicrobial resistance while preserving osteoinductive features of the matrix. We hypothesized that combining the SF matrix’s osteoconductive features and AMP release would result in a biomaterial that can prevent infections and improve bone tissue integration in orthopedic applications.

## 2. Results

### 2.1. Swelling Behavior

**Figure 1:**
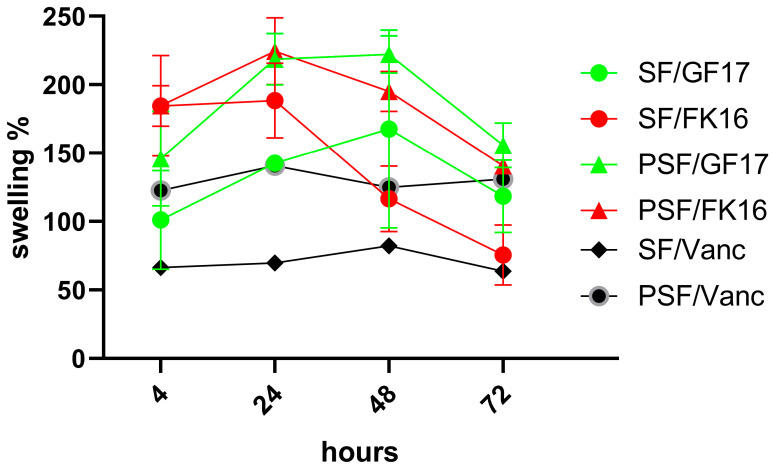
Swelling behavior of SF and PSF sponges incorporated with GF-17, FK-16, or Vancomycin over time. The swelling percentage of silk fibroin (SF) and osteoinductive peptide-enriched silk fibroin (PSF) containing antimicrobial peptides GF-17 or FK-16 was evaluated at 4, 24, 48, and 72 h. PSF sponges exhibited higher swelling capacities compared to SF sponge formulations. Among all groups, PSF/GF17 showed the highest swelling, while SF/Vanc exhibited the lowest swelling levels at all time points. Data are reported as mean ± SEM. No significant differences were detected.

The swelling behavior of four different sample formulations (SF/GF17, SF/FK16, PSF/GF17, and PSF/FK16) was assessed over 72 h, with data collected at 4, 24, 48, and 72 h (Figure 1). For each sample group, the percentage of swelling was calculated and plotted against time. After 4 h, all formulations showed an initial increase in swelling, with values ranging from approximately 100% to 160%. Among these, PSF/GF17 displayed the highest initial swelling (~160%), while SF/GF17 showed the lowest (~110%). At 24 h, all groups had reached or nearly reached their peak swelling values. PSF/GF17 and PSF/FK16 had a swelling percentage of over 200%. SF/FK16 also peaked around this time, reaching approximately 180%, while SF/GF17 showed a constant growth, reaching about 150%. All groups experienced a decrease in swelling by 48 h, except for SF/GF17, which remained at a relatively steady level (~150%). PSF/GF17 experienced a slight decrease but still exceeded 200%. In contrast, PSF/FK16 and SF/FK16 had more substantial drops, dropping to approximately 170% and 120%, respectively. After 72 h, the downward trend continued for all groups. The increased swelling percentages (160% and 140%, respectively) of PSF/GF17 and SF/GF17 show a better ability to retain water. The decrease in PSF/FK16 and SF/FK16 to ~130% and ~90%, respectively, suggests a less stable swelling behavior over prolonged periods.

Overall, despite the absence of significant differences, the PSF-based formulations (especially PSF/GF17) demonstrated superior swelling capacity and stability over time compared to their SF-based counterparts. The ability of the sponge network to absorb and retain water may be affected by the peptide type, as GF-17-incorporated samples perform better than those containing FK-16. The increased swelling observed in PSF sponges indicates an improved capability to keep water, which could enhance the local diffusion of antimicrobial peptides and facilitate nutrient exchange in vivo. The importance of this feature lies in clinical situations where maintaining moisture balance and AMP availability are essential for infection control and tissue integration. The swelling of GF-17-loaded sponges may be more elevated than that of FK-16-loaded ones, as differences in peptide–matrix interactions can affect water retention within the silk fibroin network.

### 2.2. Release Kinetics of GF-17 and FK-16 from Sponges

**Figure 2:**
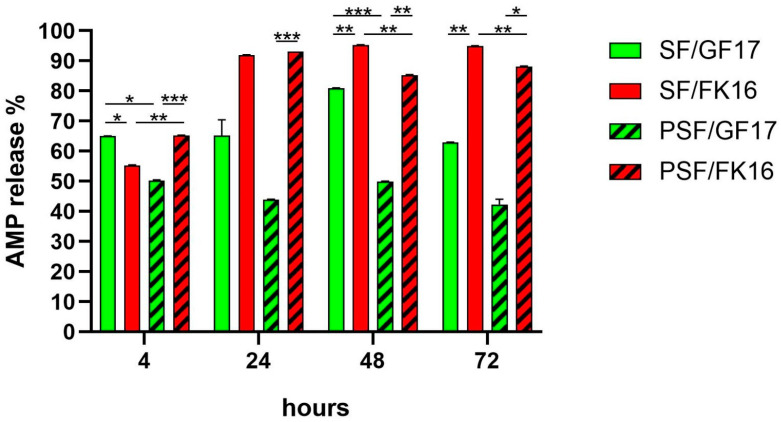
Cumulative release of AMPs from SF and PSF sponges over time. The percentage of AMP release from silk fibroin (SF) and osteoinductive peptide-enriched silk fibroin (PSF) loaded with GF-17 or FK-16 peptides was measured at 4, 24, 48, and 72 h. SF-based sponges (solid bars) and PSF-based sponges (hatched bars) showed distinct release profiles depending on the peptide incorporated. FK-16-loaded sponges exhibited significantly higher AMP release at all time points compared to their GF-17 counterparts. PSF-based formulations demonstrated a generally slower release compared to SF-based ones, particularly notable in the PSF/GF17 group. Minor decreases in cumulative release values reflect analytical variability or peptide instability, not reabsorption. Data are presented as mean ± SEM. Statistical significance between groups is * *p* < 0.05, ** *p* < 0.01, and *** *p* < 0.001.

The release profiles of AMPs GF-17 (200 µg) and FK-16 (430 µg) from both SF and PSF sponges were assessed over 72 h (Figure 2). The cumulative percentage release was quantified at 4, 24, 48, and 72 h. At 4 h, SF/GF17 showed the highest release among all groups (~65%), significantly exceeding PSF/GF17 (~50%, *p* < 0.001) and SF/FK16 (~55%, *p* < 0.05). Conversely, PSF/FK16 also exhibited a relatively high release (~66%), significantly higher than PSF/GF17 (*p* < 0.001). By 24 h, SF/FK16 and PSF/FK16 showed a marked increase in release (>90%), significantly higher than their GF17 counterparts (SF/GF17: ~65%; PSF/GF17: ~44%) with *p* < 0.001 for SF/FK16 vs. PSF/GF17 and *p* < 0.01 for PSF/FK16 vs. PSF/GF17. At 48 and 72 h, SF/FK16 maintained a high and stable release (>95%), which was significantly higher than that of other groups (*p* < 0.01). PSF/FK16 showed a similar trend, achieving a release rate of around 88–90% at later time points. In contrast, GF17-loaded sponges (both SF and PSF) exhibited a plateau or slight decrease in release percentages (SF/GF17: ~82% at 48 h and ~63% at 72 h; PSF/GF17: ~50% at 48 h and ~42% at 72 h).

Overall, FK-16 demonstrated a faster, earlier, and more stable release profile than GF-17 across both sponge types. Additionally, PSF functionalization seemed to decrease the release efficiency of GF-17 but not FK-16, indicating peptide-specific interactions with the matrix. The SF/GF17 formulation exhibited a peak release at 48 h, followed by a decline at 72 h. The PSF/GF17 formulation showed a consistently lower release across all time points. Both SF/FK16 and PSF/FK16 formulations achieved high release percentages (>85%) within 24 h and maintained this level for 72 h.

The rapid and strong antimicrobial effect of FK-16-loaded PSF sponges against MRSE isolates correlates with its higher release profile, which supports the hypothesis that early and sufficient AMP availability is essential to bactericidal activity. In contrast, GF-17’s low antibacterial performance, even with detectable release, reinforces the idea that suboptimal diffusion or retention within the PSF matrix limits its effectiveness. This suggests that optimizing peptide–matrix interactions is critical when designing AMP-based scaffolds for orthopedic infection control.

### 2.3. Comparison Between Swelling and Release Profiles of AMP-Loaded Sponges

This section describes the comparative analysis of swelling and release trends for each formulation, based on the data reported in Section 2.1 and Section 2.2

SF/GF17 release increased gradually, reaching a peak at 48 h (~81%), followed by a decline at 72 h (~63%). The swelling exhibited a parallel increase, peaking at around 220% after 48 h, before slightly decreasing. This correlation suggests that the initial hydration of the SF matrix may facilitate GF-17 diffusion, although the subsequent decline in release implies peptide depletion or potential matrix–peptide interactions that limit availability.

In contrast, PSF/GF17 demonstrated significantly lower and more stable release values (41–50%) across all time points, despite its higher swelling capacity (up to ~220% by 24–48 h). This discrepancy indicates that swelling alone does not guarantee efficient peptide diffusion when strong electrostatic interactions occur between the highly cationic GF-17 and the negatively charged osteoinductive peptides in PSF, which could explain its limited antimicrobial performance.

The SF/FK16 group showed rapid and nearly complete AMP release (~95%) within 24 h, followed by a plateau phase lasting up to 72 h. Swelling peaked at 24 h (~190%), then progressively declined. The rapid release of FK-16 aligns with its strong bactericidal effect observed in time-kill assays, suggesting that early peptide availability is critical for antimicrobial efficacy.

PSF/FK16 exhibited a comparable release pattern to SF/FK16, with high efficiency (85–93%) and minimal delay. The swelling was slightly higher than that of SF/FK16 (~230% at 24 h) but remained unchanged. This behavior indicates that PSF incorporation does not hinder FK-16 mobility and may even support a more controlled release, which contributes to the enhanced antibacterial effect seen in PSF/FK16 sponges.

These data are summarized in Table 1.

The correlation between swelling and AMP release was assessed using Spearman’s correlation analysis for all formulations (Figure S3). No significant correlation was observed for SF/GF17 (r = 0.12, *p* = 0.40), PSF/GF17 (r = –0.16, *p* = 0.36), and PSF/FK16 (r = 0.00, *p* = 0.51), while SF/FK16 showed a statistically significant negative correlation (r = –0.60, *p* = 0.21). The data indicate that AMP release is not directly influenced by swelling, which reinforces the importance of matrix–peptide electrostatic interactions in control. FK-16 and GF-17 displayed strong cationic characteristics, both of which had high isoelectric points (>10). FK-16 was predicted to have a net positive charge of +4 under experimental conditions (pH 6.5–7.0), while GF-17 displayed a slightly higher net positive charge of +5. Although this difference appears minor, it may significantly influence peptide–matrix interactions, particularly with PSF sponges that incorporate negatively charged osteoinductive peptides. The higher charge of GF-17 is likely responsible for a stronger electrostatic hold within the matrix, which limits its diffusion and reduces antimicrobial activity compared to FK-16. These observations align with the experimental findings, where GF-17 showed restricted and irregular release, whereas FK-16 achieved rapid and near-complete release within 24 h. A summary of the calculated peptide properties is provided in Figure S4.

### 2.4. Antimicrobial Activity of AMP-Loaded SF and PSF Sponges Against MRSE Strains

The antibacterial efficacy of silk fibroin (SF) and osteoinductive peptide-enriched silk fibroin (PSF) sponges loaded with either GF-17 (200 or 400 µg/sponge) or FK-16 (430 or 860 µg/sponge) was evaluated over 7 days against two clinical methicillin-resistant *Staphylococcus epidermidis* (MRSE) isolates, GOI-1153754-03-14 (GOI) and OGSA-Sep145, and ATCC35984 (LGC). Bacterial viability was assessed by colony-forming unit (CFU/mL) counts at time 0, 24, 48, 72 h, and 7 days (T0, T1, T2, T3, and T7, respectively).

#### 2.4.1. GF-17 200 µg/Sponge and FK-16 430 µg/Sponge

**Figure 3:**
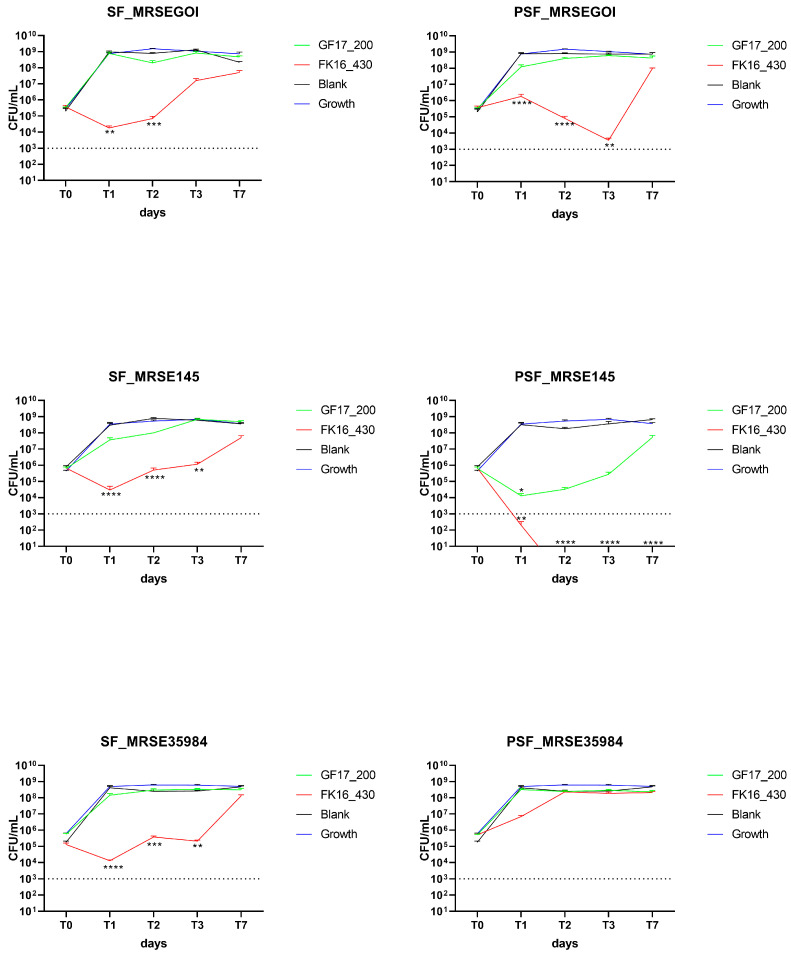
Antimicrobial activity of AMP-loaded SF and PSF sponges at FK-16 430 µg/sponge and GF-17 200 µg/sponge against MRSE isolates. Measurements (CFU/mL) were recorded at T0, T1, T2, T3, and T7. FK-16-loaded sponges showed greater antibacterial efficacy than GF-17, particularly when delivered via the PSF matrix. PSF/FK16 resulted in a rapid reduction in bacterial counts, often reaching undetectable levels between T1 and T7. SF/FK16 also exhibited inhibitory activity, though to a lesser extent. ATCC35984 responded similarly in the SF compound, but not in PSF. Limit of detection (LoD): 10^3^ CFU/mL (dash line). Data are reported as mean ± SEM. * *p* < 0.05, ** *p* < 0.01, *** *p* < 0.001, **** *p* < 0.0001 compared to blank. Only *p*-values of reductions ≥ 3 log_10_ CFU/mL are shown. MRSEGOI, MRSE GOI1153754-03-14; MRSE145, MRSE OGSA-Sep145; MRSE35984, MRSE ATCC35984. Growth, growth curves of free bacteria (blue line).

In the case of MRSE GOI, FK-16-loaded sponges overall exhibited a reduction in CFU/mL compared to GF-17-loaded sponges and blank controls (Figure 3). SF/GF17 maintained high bacterial loads (~10^9^ CFU/mL) throughout the experiment, whereas SF/FK16 induced a significant decrease in CFU already at T1 (*p* < 0.01), maintained up to T2, and partial regrowth was observed at T3. A similar pattern was observed on MRSE GOI with PSF sponges, where PSF/FK16 caused a significant bacterial reduction starting from T1 (*p* < 0.0001), maintained up to T3, followed by regrowth at T7. Conversely, the PSF/GF17 treatment exhibited no antibacterial activity, with bacterial growth from T1 onwards. The antimicrobial effect of FK-16-loaded PSF sponges against MRSE isolates correlates with its higher release profile, supporting the hypothesis that early and sufficient AMP availability is essential for bactericidal activity. In contrast, GF-17’s low antibacterial performance, despite detectable release, reinforces the idea that suboptimal diffusion or retention within the PSF matrix limits its effectiveness. This suggests that AMP-based scaffolds for orthopedic infection control need to be optimized to optimize peptide–matrix interactions.

Regarding MRSE OGSA-Sep145, FK-16 exhibited antibacterial efficacy, particularly in PSF scaffolds, where bacterial counts fell below detection limits starting from T2 and remained undetectable through T7 (*p* < 0.0001). SF/FK16 caused a significant reduction in CFU from T1 (*p* < 0.0001), although a slight increase was noted at T7. GF-17 resulted in a moderate decrease across both SF and PSF scaffolds, with CFU levels consistently ranging between 10^7^ and 10^8^ CFU/mL.

On MRSE ATCC35984, SF/FK16 showed antimicrobial activity comparable to the other two strains; however, no strong effect was observed for PSF/FK16.

Time-kill assays using Vancomycin showed a reduction below the limit of detection from T1 in the two clinical isolates (MRSE GOI and OGSA-Sep145), while on the ATCC35984, the PSF formulation dropped to 10^3^ CFU/mL at T1 and below the limit of detection at T2 (*p* < 0.0001, Figure S1).

#### 2.4.2. GF-17 400 µg/Sponge and FK-16 860 µg/Sponge

**Figure 4:**
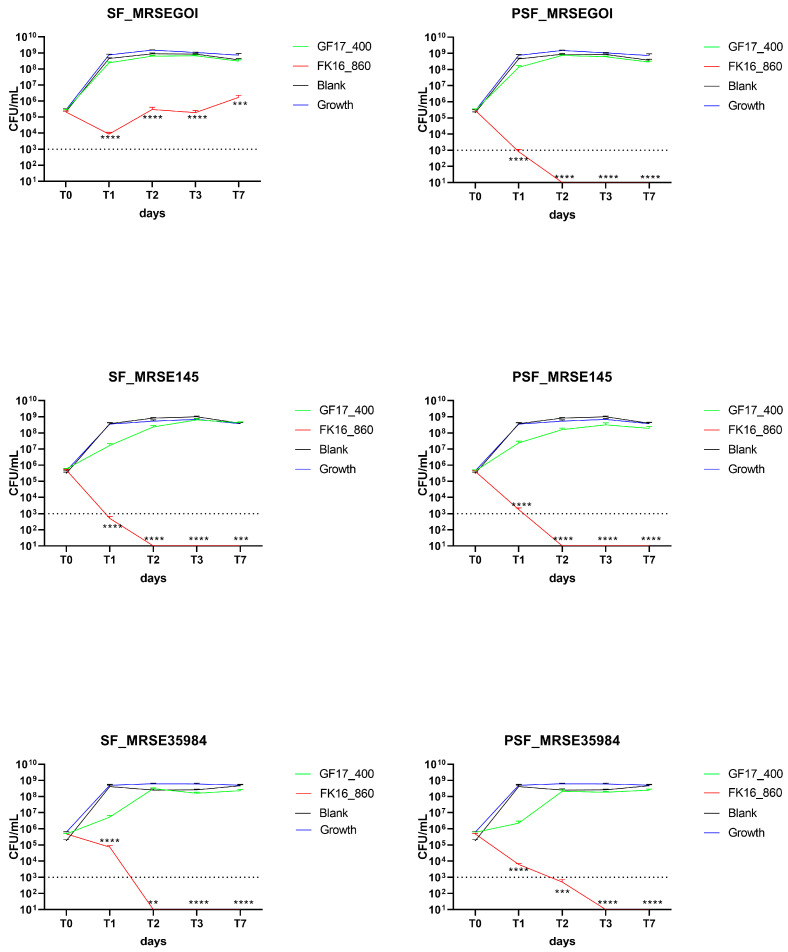
Antimicrobial activity of AMP-loaded SF and PSF sponges at FK-16 at 860 µg/sponge and GF-17 at 400 µg/sponge against MRSE isolates. Measurements (CFU/mL) were recorded at T0, T1, T2, T3, and T7. FK-16-loaded sponges showed greater antibacterial efficacy than GF-17, particularly when delivered via the PSF matrix. PSF/FK16 resulted in a rapid reduction in bacterial counts, often reaching undetectable levels between T1 and T7. SF/FK16 also exhibited inhibitory activity, though to a lesser extent. Limit of detection (LoD): 10^3^ CFU/mL (dash line). Data are reported as mean ± SEM. ** *p* < 0.01, *** *p* < 0.001, **** *p* < 0.0001 compared to blank. Only *p*-values of reductions ≥ 3 log_10_ CFU/mL are shown. MRSEGOI, MRSE GOI1153754-03-14; MRSE145, MRSE OGSA-Sep145; MRSE35984, MRSE ATCC35984. Growth, growth curves of free bacteria (blue line).

Double AMP doses were tested to determine if increased loading could improve bactericidal activity (Figure 4). In the case of MRSE GOI, SF/FK16 significantly reduced bacterial counts at T1 (*p* < 0.0001), with partial regrowth observed at T7 (*p* < 0.001). PSF/FK16 showed superior effectiveness, clearing bacteria completely through T1 and maintaining undetectable CFU levels through T7 (*p* < 0.0001). GF-17 failed to reach the bactericidal thresholds.

Similarly, for MRSE OGSA-Sep145, SF/FK16 led to a rapid decrease in CFU, with bacterial loads approaching the detection limit by T1 and remaining suppressed (*p* < 0.001). PSF/FK16 completely eradicated bacteria from T2 onwards (*p* < 0.0001). There was no significant activity of GF-17, only slight decreases. With MRSE ATCC35984, FK-16 maintained strong antibacterial activity, with a total eradication of the bacterial load from T2 using the SF formulation (*p* < 0.01) and the PSF formulation (*p* < 0.001). Similar to the other strains, GF-17 remained largely ineffective despite the higher concentration.

### 2.5. Antimicrobial Activity of AMP-Loaded SF and PSF Sponges Against MRSA Strains

The antibacterial efficacy of silk fibroin (SF) and osteoinductive peptide-enriched silk fibroin (PSF) sponges loaded with either GF-17 (200 µg/sponge) or FK-16 (430 µg/sponge) was evaluated over 7 days, as previously described, against MRSA 20/24, MRSA 32/24, and ATCC43300.

**Figure 5:**
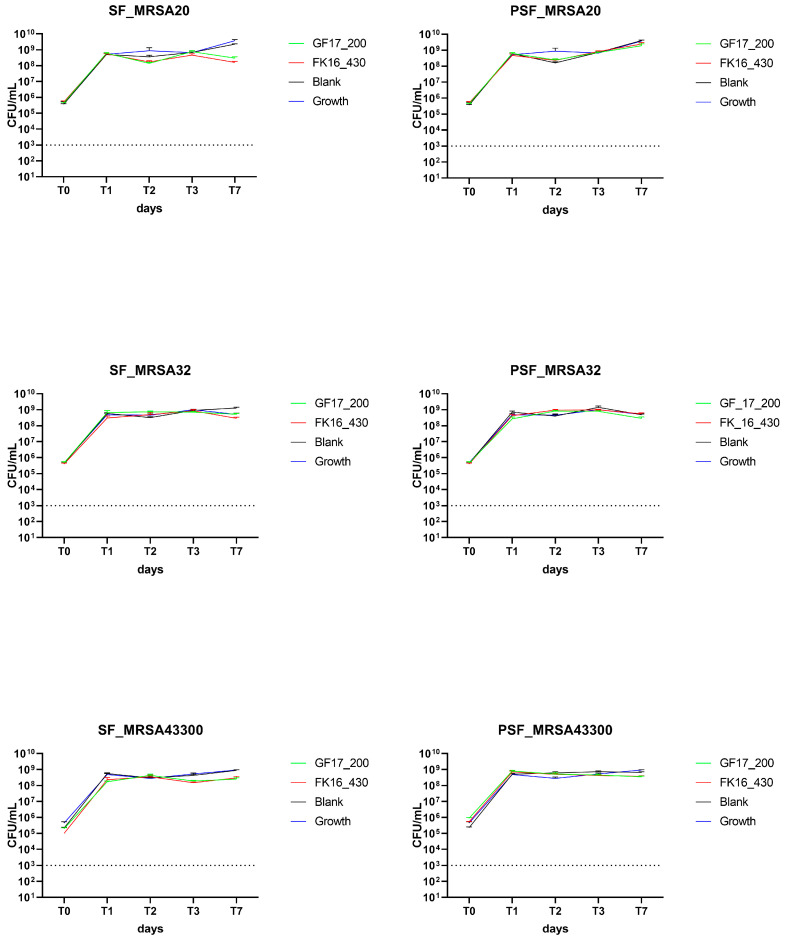
Antimicrobial activity of AMP-loaded SF and PSF sponges (FK-16 at 430 µg/sponge and GF-17 at 200 µg/sponge) against MRSA. Bacterial viability (CFU/mL) over 7 days for clinical *Staphylococcus aureus* MRSA20, MRSA32, and ATCC43300 treated with SF or PSF, loaded with either GF-17 or FK-16. CFU counts were recorded at time points T0, T1, T2, T3, and T7. Both AMP-loaded sponges failed to significantly reduce bacterial load compared to controls (blank), indicating limited or no antimicrobial activity of GF-17 and FK-16 against these MRSA strains under the tested conditions. Limit of detection (LoD): 10^3^ CFU/mL (dash line). Data are reported as mean ± SEM. Only *p*-values of reductions ≥ 3 log_10_ CFU/mL are shown. MRSA20, MRSA 20/24; MRSA32, MRSA 32/24, MRSA43300, ATCC43300. Growth, growth curves of free bacteria (blue line).

AMP-loaded sponges containing either FK-16 (430 µg/sponge) or GF-17 (200 µg/sponge) failed to exhibit significant antibacterial effects compared to blank (unloaded) controls on the three isolates tested. In all tested conditions, including both SF and PSF matrices, the growth of bacteria was rapid and maintained throughout the experimental timeline (Figure 5).

The results indicate that MRSA strains were not further tested with higher concentrations of AMPs. The lack of antimicrobial activity of both FK-16 and GF-17 against MRSA isolates under the tested conditions suggests strain-specific differences in susceptibility to AMPs. These findings align with previous literature reporting higher resistance of MRSA to cathelicidin-derived peptides, likely due to membrane composition and proteolytic defense mechanisms. This highlights that AMP-functionalized scaffolds may require either higher peptide loading or synergistic approaches (e.g., AMP combined with conventional antibiotics) to overcome MRSA-associated challenges in orthopedic infections.

Time-kill assays using Vancomycin showed a reduction below the limit of detection from T1 in the two clinical isolates (MRSA 20/24 and MRSA 32/24), while on the ATCC43300, both formulations dropped to 10^3^ CFU/mL at T1 and below the limit of detection at T2 (*p* < 0.0001, Figure S2).

### 2.6. Linking AMP Release to Antimicrobial Effectiveness Against MRSE and MRSA

Two sponge formulations, silk fibroin (SF) and osteoinductive peptide-silk fibroin (PSF), were used to interpret the release kinetics of GF-17 and FK-16, which are presented in Table 2. The concentration of AMPs was set at 200 µg for GF-17 and 430 µg for FK-16 per sponge.

AMP release data showed that GF-17 had a maximum release of 81% at 48 h in SF and 50% in PSF; nevertheless, it was ineffective in reducing bacterial growth against both MRSA and MRSE strains. Conversely, FK-16 demonstrated significantly higher release efficiencies—95% in SF and 93% in PSF—particularly in samples showing antimicrobial activity against MRSE strains (Mann–Whitney U test, *p* = 0.0286; exact 97.14% confidence interval for the difference: 4.00 to 51.00). Spearman’s rank correlation showed a significant correlation between percentage release and antimicrobial activity (r = 0.8729, exact *p* = 0.0286).

## 3. Discussion

In this study, we developed and evaluated SF- and PSF-based sponges as local delivery systems for the antimicrobial peptides FK-16 and GF-17, targeting implant-associated infections caused by methicillin-resistant *Staphylococci*. Our research shows that FK-16, especially when combined with osteoinductive peptide-enriched silk fibroin (PSF) sponges, has a powerful and persistent bactericidal effect against clinical MRSE strains. Regardless of the formulation, GF-17 had limited efficacy, and neither peptide demonstrated significant antimicrobial activity against MRSA isolates.

This difference highlights the critical role of peptide–matrix interactions in determining therapeutic outcomes. FK-16’s rapid release correlated with efficient bacterial clearance, while GF-17’s limited diffusion, particularly in PSF matrices, suggests strong electrostatic retention due to its higher positive charge. These findings underscore that matching AMP physicochemical properties with scaffold chemistry is essential for optimizing drug delivery.

The antimicrobial efficacy of the AMPs (FK-16 and GF-17) used in this study has previously been validated in vitro against both planktonic and biofilm-associated forms of *S*. *aureus* and *S. epidermidis*, including methicillin-resistant strains. Their low cytotoxicity and high selectivity index were confirmed in previous work through MIC/MBC determination, hemolysis, and biofilm disruption assays [7]. These findings support the use of AMP concentrations in the present study and their therapeutic potential in implant-related infections. Additionally, the use of PSF as a delivery matrix is supported by previous research in which PSF sponges demonstrated osteoinductive properties and promoted bone ingrowth when used in combination with trabecular titanium implants in a large animal model [13]. Moreover, the PSF matrix has been successfully functionalized with antibiotic-loaded nanoparticles, demonstrating dual osteoregenerative and antimicrobial activity in a rat model of *S. epidermidis*-induced infected nonunions [15].

The observed differences between FK-16 and GF-17 emphasize the need for rational design in AMP-loaded scaffolds. GF-17’s limited activity even at higher concentrations suggests that increasing AMP dosage alone may not be enough to overcome strong matrix-binding interactions. This highlights that scaffold charge density and peptide characteristics must be co-optimized during material design.

The physicochemical interaction with the SF and PSF matrices is the primary reason for the different antimicrobial outcomes between FK-16 and GF-17. FK-16 showed a faster and more complete release from both SF and PSF sponges (>85% within 24–48 h), which correlates with robust and stable bactericidal activity against MRSE clinical isolates. GF-17, on the other hand, demonstrated lower release efficiencies—particularly from PSF sponges—suggesting stronger electrostatic interactions with the negatively charged PSF. A retention effect may be caused by the higher net positive charge of GF-17 at physiological pH, which may limit its diffusion and antimicrobial performance. This seemingly small difference may significantly impact peptide–matrix interactions, particularly in PSF sponges enriched with negatively charged osteoinductive peptides. The stronger electrostatic interaction between GF-17 and the PSF matrix likely leads to a decrease in its release and efficacy on bacterial strains, as it is more tightly held within the scaffold structure. This hypothesis is consistent in its low release values observed for PSF/GF17 compared to other formulations, despite similar or higher swelling capacities. Similar observations have been reported with other cationic AMPs, where scaffold–peptide interactions critically affect bioavailability and therapeutic outcomes [9,19]. The importance of tailoring the carrier matrix composition to the physico-chemical characteristics of the loaded AMPs is underscored by our findings in order to achieve optimal therapeutic release. The antimicrobial inefficacy of GF-17 observed in our system, despite detectable release, could be attributed to peptide–matrix electrostatic interactions, as previously described in studies examining positively charged peptides retained in negatively charged hydrogels or scaffolds [20]. The water uptake properties of the scaffolds further confirm the role of matrix hydration in facilitating AMP diffusion. Compared to SF sponges, PSF sponges have a higher swelling capacity, with PSF/GF17 exhibiting the highest water retention (220% at 24–48 h). Swelling alone was sufficient for AMP diffusion when matrix–peptide affinity is high; however, it did not result in effective GF-17 release, which suggests that matrix–peptide affinity alone is not sufficient. This interpretation is statistically supported by the correlation analysis, which showed no significant relationship between swelling and AMP release for SF/GF17, PSF/GF17, and PSF/FK16. These findings confirm that swelling alone is not a reliable predictor of AMP release. Instead, peptide–matrix electrostatic interactions play a dominant role, particularly for GF-17, which remained strongly retained despite higher swelling values. The suggestion is that scaffold design should prioritize chemical compatibility over hydration properties in optimizing controlled AMP delivery. FK-16’s swelling behavior positively correlates with fast peptide release, which promotes efficient antimicrobial action. The interplay between swelling, release, and antimicrobial efficacy reflects a multifactorial system where matrix composition, AMP properties, and environmental conditions must be co-optimized. Recent silk-based delivery systems that combine LL-37 with osteoconductive or immunomodulatory indications have shown a similar synergistic behavior [17]. Theory suggests that the enhanced swelling of PSF sponges promotes peptide diffusion and tissue integration. Swelling was not enough to guarantee GF-17 bioavailability, indicating that peptide–matrix affinity can override hydration dynamics. PSF/FK16’s superior antimicrobial performance is likely due to its high swelling and minimal retention. AMP-functionalized sponges have several advantages over conventional antibiotic-loaded scaffolds. FK-16-loaded PSF sponges demonstrated efficacy comparable to vancomycin-loaded scaffolds in vitro [14]. Additionally, PSF has been previously shown to support osteointegration in vivo, suggesting potential dual functionality in bone regeneration and infection control [13,15]. This dual role is particularly important when dealing with infected bone defects or revision arthroplasty. Silk fibroin/poloxamer composites have been successfully applied to deliver drugs to MRSA-infected skin wounds, promoting healing and microbial clearance [21]. This confirms that SF is a versatile platform for delivering AMP to different tissue types. The potential of electrospun nanofiber systems loaded with AMP analogues (17BIPHE2) in wound infection models is being explored [22]. Compared to nanofibers, our sponge system offers greater capacity for peptide loading and potentially better mechanical integration with orthopedic implants.

In our study, PSF/FK16 showed the highest and longest antibacterial activity in both clinical MRSE strains and ATCC35984, demonstrating the enhanced peptide release and bioactivity provided by the PSF carrier coupled with FK-16. The different response to ATCC35984 suggests that the response to AMPs could be based on both bacteria and dose. Overall, FK-16 at 860 µg, particularly when delivered via PSF sponges, achieved rapid bactericidal activity against all MRSE strains tested, whereas GF-17 displayed limited efficacy even at increased doses. This result aligns with findings from Mishra and Wang [23], who showed that FK-16 immobilized on titanium surfaces effectively disrupts Gram-positive biofilms, suggesting that both surface-tethered and sustained-release formats can offer localized antimicrobial protection in orthopedic settings.

Despite these promising results, our AMP-functionalized sponges were not successful in combating MRSA isolates under the tested conditions, which confirmed previous findings of AMP susceptibility based on species [21]. Higher doses or synergistic combinations (e.g., AMP + antibiotic) might be required. The absence of effects between SF and PSF matrices indicates that the carrier composition or AMP dosage was not sufficient to overcome the inherent resistance of MRSA20 and MRSA32. These data confirm the species-dependent variability in AMP susceptibility, with MRSA isolates showing a markedly higher tolerance to FK-16 and GF-17 under the tested experimental parameters. The literature suggests that AMP susceptibility can be genus or species-specific [6]. The failure of FK-16 and GF-17 to reduce MRSA viability, even at elevated doses, suggests intrinsic resistance mechanisms in these strains, possibly related to their membrane composition or proteolytic environments. The data show how crucial it is to integrate release behavior with functional outcomes in the design of AMP-based biomaterials.

The present study focused on testing only two MRSE and two MRSA clinical isolates, along with reference strains. Although the results were consistent, broader testing is needed to generalize efficacy. The decrease in GF-17 release at later time points may be caused by peptide degradation; however, it was not measured directly, which is a drawback of this study. Enzymatic stability assays could reveal the impact of proteolysis on AMP performance. Although FK-16 and GF-17 have been shown to inhibit biofilm formation in previous studies [7], the focus of this work was on planktonic bacteria. AMP-loaded sponges have yet to be determined to disrupt established biofilms in situ. Despite promising in vitro results against MRSE, the lack of activity against MRSA suggests species-specific differences in AMP susceptibility, likely due to MRSA’s altered cell membrane charge and proteolytic defenses. Future strategies may involve AMP-antibiotic combinations or structural modifications to enhance activity against highly resistant pathogens.

This study shows that AMP-loaded silk fibroin scaffolds, particularly FK-16 in PSF matrices, can serve as versatile biomaterials for orthopedic infection control. The integration of antimicrobial and osteoinductive functions in a single platform is a promising direction towards reducing infection rates and improving implant integration. Taken together, these findings reinforce the rationale for using AMP-loaded PSF scaffolds as a bioactive platform for orthopedic applications, especially in high-risk scenarios such as infected bone defects or revision arthroplasty. Future in vivo studies will provide further insight into the translational potential of this system. Overall, tailoring AMP release behavior by adjusting matrix composition, charge, and structure represents a key step towards the clinical translation of multifunctional scaffolds that combine infection control with osteoinductive properties.

## 4. Materials and Methods

### 4.1. Sponge Preparation and AMP Loading

Standard Fmoc-based solid-phase synthesis on Rink amide MBHA resin was employed to synthesize AMPs, utilizing a 5-fold excess of Fmoc-protected amino acids coupled through DCC/HOBt. Cleavage and deprotection were performed with TFA/H_2_O/triisopropylsilane (90:5:5, *v*/*v*/*v*), followed by ether extraction. The purification of crude peptides was performed through reverse-phase HPLC (Vydac C18 column) with the use of a water/acetonitrile gradient with 0.1% TFA. Silk fibroin (SF) fibers were obtained by degumming *Bombyx mori* cocoons through autoclave treatment in deionized water at 120 °C for 20 min. To eliminate the residue of sericin, the fibers were thoroughly washed with hot water. The purified fibroin was dissolved in a 9.3 M lithium bromide (LiBr) solution (Merck, Darmstadt, Germany) with gentle stirring at 60 °C for 3 h, yielding a 10% *w*/*v* solution. This solution was subsequently diluted in a 2:1 ratio with distilled water, filtered, and placed into a cellulose dialysis membrane (molecular weight cutoff: 14.000; Sigma–Aldrich, Milan, Italy). Dialysis continued until complete salt removal was confirmed, resulting in an SF solution with an approximate concentration of 3.3% *w*/*v* [14]. To isolate the osteoinductive Cs peptide fraction, the SF solution was enzymatically hydrolyzed using α-chymotrypsin (Sigma–Aldrich, Milan, Italy) at an enzyme-to-substrate ratio of 1:100. The reaction proceeded for 24 h at 37 °C. Following incubation, the mixture was centrifuged at 7400× *g* for 20 min to separate the insoluble fraction. The supernatant, containing the target anionic peptides, was then freeze-dried to obtain the Cs peptide fraction in powdered form. Cs powder has been dissolved in a minimum amount of water and added to SF solution, generating Cs peptide-enriched SF (PSF) [13,14]. Following lyophilization of the SF and PSF solutions, each sponge—SF and PSF—with an average dry weight of approximately 2.4 mg and 2.9 mg, respectively, was functionalized by applying aqueous solutions (10 µL) containing defined amounts of antimicrobial peptides (AMPs), namely FK-16 (430 or 860 µg per sponge) or GF-17 (200 or 400 µg per sponge). These loading amounts correspond to peptide-to-sponge ratios of approximately 150–370 µg/mg. Although these values may appear close to or exceeding 100% when expressed relative to sponge mass, it is important to clarify that they refer to the mass of peptide loaded per milligram of dry scaffold, not as a percentage of sponge composition. This distinction reflects the high loading capacity of the porous silk matrix and ensures uniform peptide distribution on the scaffold surface without oversaturation. The quantities of FK-16 and GF-17 loaded onto the silk fibroin sponges were determined using a two-step approach. Initially, concentrations were selected according to a previous study, assessing the antimicrobial efficacy of the present AMPs as a suspension [7]. To achieve effective antimicrobial activity in the sponge matrix, the concentrations of FK16 and GF17 were increased to the following values: FK-16 at 430 or 860 µg/sponge, and GF-17 at 200 or 400 µg/sponge. Uniform peptide distribution was ensured by applying the solutions directly onto the scaffold surface. As a positive control, SF and PSF were loaded with Vancomycin at concentrations equivalent to 10× EUCAST breakpoints [24] and tested using the time-kill assay to confirm the intrinsic ability of the silk fibroin scaffold to deliver antibiotics. Vancomycin was included as a reference control to validate the functionality of the delivery system and to ensure that observed effects were due to peptide properties rather than limitations of the silk-based matrices.

### 4.2. Water Uptake Test

Water uptake analysis was used to assess the swelling behavior of SF and PSF sponges loaded with AMPs (FK-16 at 430 µg or GF-17 at 200 µg) or Vancomycin. An analytical balance was used to accurately weigh dry sponges (Wd). The simulated physiological conditions involved immersing each sample in 1 mL of phosphate-buffered saline (PBS, pH 7.4) and incubating it at 37 °C. After incubation times of 4, 24, 48, and 72 h, the samples were carefully removed from PBS, gently blotted with filter paper to eliminate surface water without squeezing, and immediately weighed to obtain wet weight (Ww). The water uptake (swelling ratio) was calculated using the following formula:

Swelling (%) = (Ww − Wd)/Wd × 100

### 4.3. Release Studies

This study investigates the release profiles of AMPs (FK-16 at 430 µg or GF-17 at 200 µg) from the two types of sponges: SF and PSF. For each AMP (GF17 and FK16), two sponges were placed in 200 µL of Milli-Q water per vial, resulting in a total of two vials per condition. The vials were incubated at 37 °C. At predetermined time points (4, 24, 48, and 72 h), aliquots of 10 µL from each vial were diluted with 180 µL of water. This process was consistent across all samples. Reverse-phase High-Performance Liquid Chromatography (HPLC) was used to analyze the diluted samples with UV detection at 230 nm and an injection volume of 10 or 20 µL. The column used was Aeris Peptide XB-C18, 3.6 µm, 2.1 × 150 mm, and the mobile phase transitioned from H_2_O/ACN + TFA 85:15 to 30:70 over 38 min at 40 °C. Based on a standard calibration curve, the area under the curve (AUC) from the HPLC chromatograms was used to quantify the amount of AMP released. The theoretical AMP content per injection (THEOR AMP) was calculated based on the initial loading and dilution factors.

To assess the efficiency of AMP release relative to the theoretical maximum, the following calculation was performed and normalized, allowing for direct comparison across different formulations and time points:

% Release = (μg Released/THEOR AMP) × 100

The free peptide analysis tool PepCalc (https://pepcalc.com/) was used for preliminary in silico calculations to investigate the differences in release behavior between FK-16 and GF-17. These simulations were based on the amino acid sequences of the two peptides and provided estimates of their physicochemical properties, including the isoelectric point (pI) and net charge at physiological pH.

### 4.4. Bacterial Strains and Culture

A subset of clinically relevant methicillin-resistant *Staphylococcus* strains was used for antimicrobial testing, including two clinical isolates of methicillin-resistant *Staphylococcus epidermidis* (MRSE) and two clinical isolates of methicillin-resistant *Staphylococcus aureus* (MRSA). The MRSE (GOI1153754-03-14 and OGSA-Sep145) and the MRSA (MRSA 20/24 and MRSA 32/24) were selected from the IRCCS Ospedale Galeazzi—Sant’Ambrogio of Milan (Italy) strain collection. MRSE ATCC35984 and MRSA ATCC43300 (LGC Standards, Milan, Italy) were tested in parallel with the clinical isolates. All bacterial strains were stored at −80 °C, and working stocks were maintained at −20 °C. For experimental use, bacteria were resuscitated in Brain Heart Infusion (BHI; Millipore, Milan, Italy) broth at 37 °C for 18 h. To ensure viability and purity during the experimental procedures, cultures were routinely maintained on tryptic soy agar (TSA; Millipore, Milan, Italy).

### 4.5. Time-Kill Assays

Time-kill assays were conducted with the bacterial strains mentioned to evaluate the antimicrobial activity of antibiotic- or AMP-loaded SF- and PSF-sponges. Briefly, 100 µL of bacterial suspension (~1 × 10^6^ CFU/mL) was added to Eppendorf tubes containing 900 µL of BHI broth. One sponge (SF or PSF, loaded with Vancomycin, GF-17, or FK-16) was added per tube, according to the tested condition. Each antibiotic formulation (SF/Van, PSF/Van) was tested in duplicate at concentrations 10× EUCAST breakpoints against the aforementioned bacterial strains, all of which were susceptible to Vancomycin. Each AMP formulation—SF/GF17, SF/FK16, PSF/GF17, and PSF/FK16—was tested at two concentrations (GF-17: 200 and 400 µg/sponge; FK-16: 430 and 860 µg/sponge) in duplicate. The bacterial viability of tubes was assessed at 1, 2, 3, and 7 days after incubation at 37 °C. At each time point, 10 µL of the bacterial suspension was serially diluted and plated (10 µL drop-plating) onto tryptic soy agar (TSA; Millipore, Milan, Italy). Plates were incubated for 24 h at 37 °C, and colonies were counted to calculate the viable bacterial load, expressed as log_10_ CFU/mL. A reduction of ≥3 log_10_ CFU/mL from the initial inoculum was considered indicative of bactericidal activity. Two positive controls consisting of bacterial suspension in BHI without sponges and SF- or PSF-unloaded sponges were included in each experiment. Additionally, vancomycin-loaded SF and PSF sponges were included as positive controls to confirm the reliability of the assay. Unloaded SF/PSF controls provided a reference for bacterial growth in the presence of the unloaded materials, while growth curves showed the normal bacterial growth over time.

### 4.6. Statistical Analysis

Statistical comparisons for swelling and AMP release profile data were performed on GraphPad Prism v. 8.0, using the two-way analysis of variance (ANOVA), followed by Tukey’s post hoc test for multiple comparisons. Kruskal-Wallis with Dunn’s multiple comparison was used to compare treatments at each time point of the time-kill assays. This approach allowed for the evaluation of the effects of both formulation type and time, as well as their interaction. Results are presented as mean ± standard error (SEM). A *p*-value <0.05 was considered statistically significant. The correlations between swelling and release, and AMP release profiles and the antimicrobial activity of SF and PSF sponges were assessed using Spearman’s rank correlation and further confirmed with the Mann–Whitney U test.

## Figures and Tables

**Table 1:** Summary of swelling and release behavior of SF- and PSF-based sponges functionalized with AMPs (GF-17 and FK-16).

Formulation	Swelling Trend	Release Trend	Interpretation
SF/GF17	Peaks at 48 h (~220%)	Peaks at 48 h, then declines	Swelling possibly enhances the release initially; later drop may reflect depletion
PSF/GF17	High swelling (~220%)	Constant low release (41–50%)	Swelling is not sufficient; likely peptide retention or interactions
SF/FK16	High at 24 h, decreases	Rapid release, plateau	Early swelling likely supports burst release
PSF/FK16	Highest swelling (~230%)	Rapid release, plateau	Swelling may promote and support release

**Table 2:** Correlation between AMP release levels and antimicrobial activity of SF and PSF sponges. Spearman’s rank correlation (r = 0.8729, exact *p* = 0.0286) and Mann–Whitney U test, * *p* < 0.05.

AMP/Formulation	Release (%)	MRSE Activity	MRSA Activity
SF/GF17	70–81	None	None
PSF/GF17	44–50	None	None
SF/FK16	92–95 *	Yes	None
PSF/FK16	85–93 *	Yes	None

## Data Availability

The original data presented in the study are openly available in Zenodo at DOI 10.5281/zenodo.16742951.

## Supplementary Materials

The following supporting information can be downloaded at: https://www.mdpi.com/article/10.3390/ijms26167775/s1.
