# Supplementary material for: Development of Silk Fibroin-Based Sponges Loaded with LL-37-Derived Peptides for the Control of Orthopedic Infections
Source: Int J Mol Sci. 2025 Aug 12;26(16):7775. doi: 10.3390/ijms26167775 (PMC12386548; doi:10.3390/ijms26167775)
Supplement: Supplementary file 1 [file ijms-26-07775-s001.zip › ijms-3782367-supplementary.pdf]

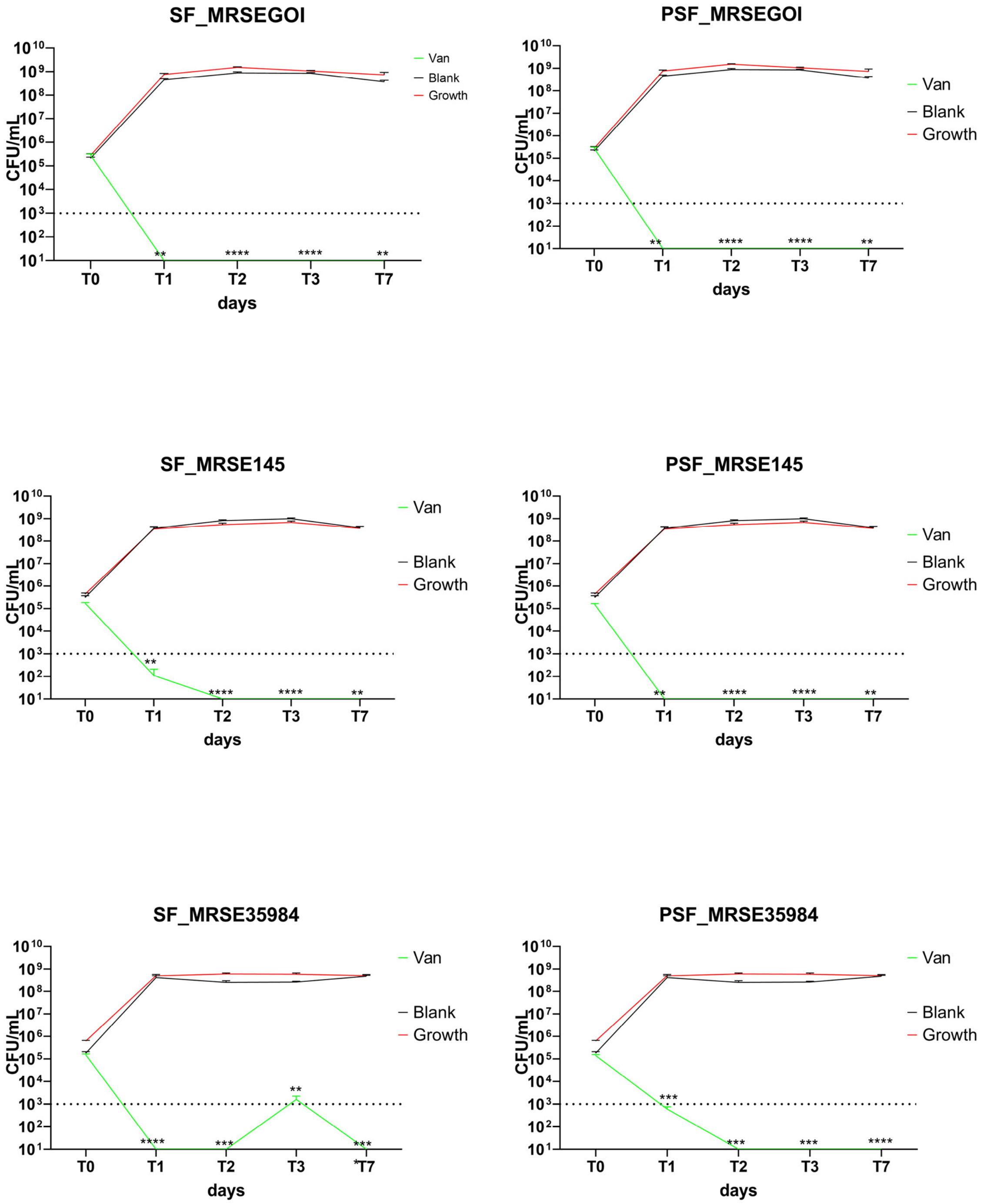

Figure S1: Antimicrobial activity of Vancomycin-loaded SF and PSF sponges against MRSE.

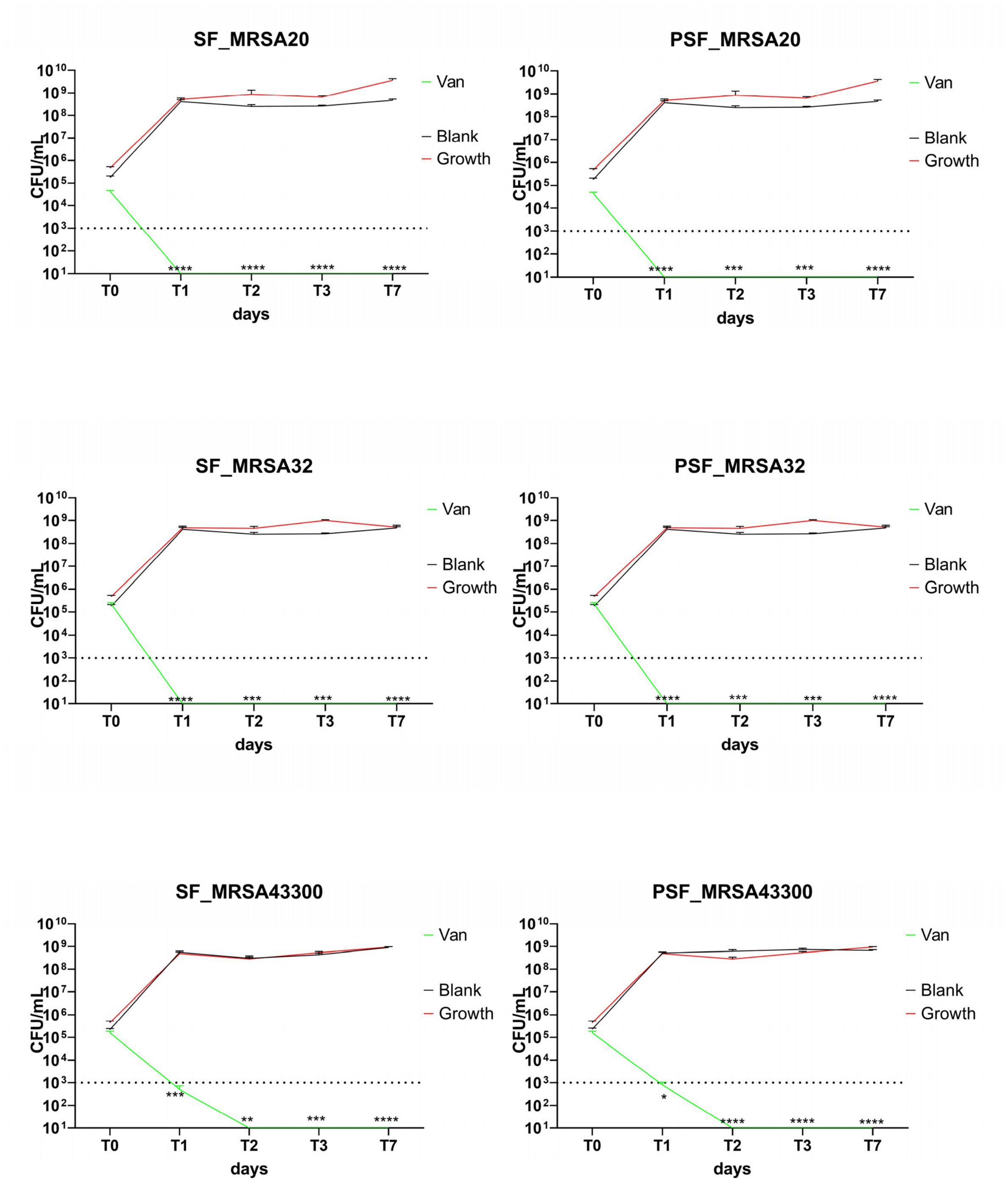

Figure S2: Antimicrobial activity of Vancomycin-loaded SF and PSF sponges against MRSA.

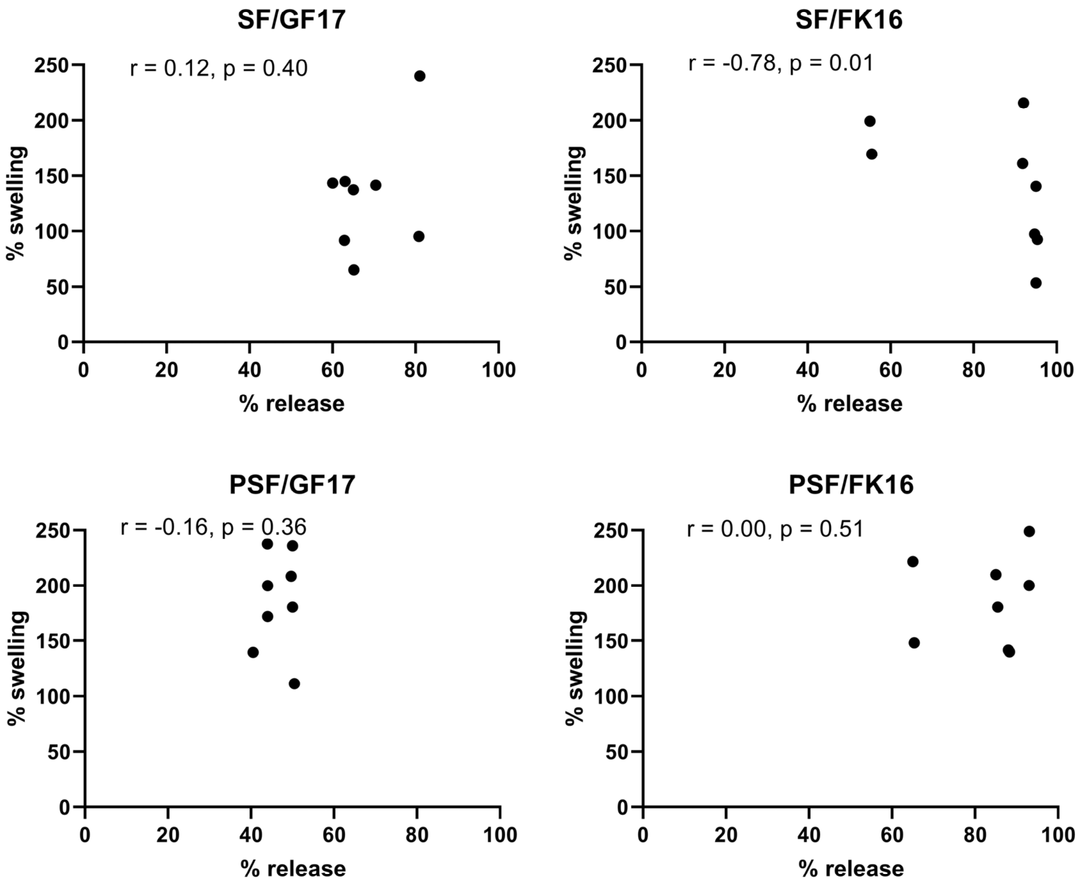

Figure S3: Scatter plots of the correlation between swelling and release of AMP-loaded SF and PSF.

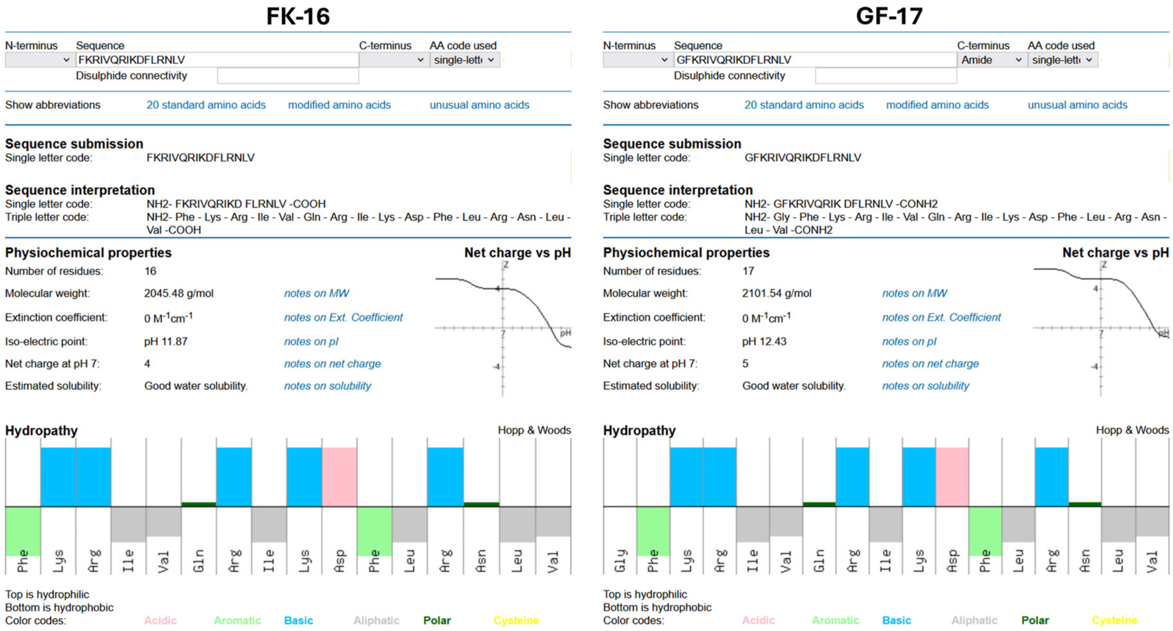

Figure S4: Physico-chemical properties of FK-16 and GF-17 predicted with PepCalc (https://pepcalc.com/).
